# Travelling Wave Ion Mobility-Derived Collision Cross Section for Mycotoxins: Investigating Interlaboratory and Interplatform Reproducibility

**DOI:** 10.1021/acs.jafc.0c04498

**Published:** 2020-09-01

**Authors:** Laura Righetti, Nicola Dreolin, Alberto Celma, Mike McCullagh, Gitte Barknowitz, Juan V. Sancho, Chiara Dall’Asta

**Affiliations:** † Department of Food and Drug, University of Parma, Viale Delle Scienze 17/A, I-43124 Parma, Italy; ‡ Waters Corporation, Altrincham Road, SK9 4AX Wilmslow, United Kingdom; § Environmental and Public Health Analytical Chemistry, Research Institute for Pesticides and Water, University Jaume I, Avda. Sos Baynat s/n, E-12071 Castellón, Spain

**Keywords:** mycotoxins, food residues, travelling wave ion mobility separation, CCS database, interlaboratory comparison, interplatform

## Abstract

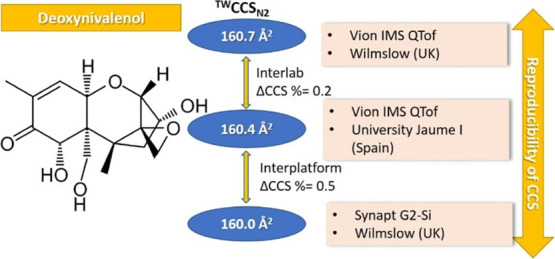

Parent and modified mycotoxin analysis remains a challenge because of their chemical diversity, the presence of isomeric forms, and the lack of analytical standards. The creation and application of a collision cross section (CCS) database for mycotoxins may bring new opportunities to overcome these analytical challenges. However, it is still an open question whether common CCS databases can be used independently from the instrument type and ion mobility mass spectrometry (IM-MS) technologies, which utilize different methodologies for determining the gas-phase mobility. Here, we demonstrated the reproducibility of CCS measurements for mycotoxins in an interlaboratory study (average RSD 0.14% ± 0.079) and across different traveling wave IM-MS (TWIMS) systems commercially available (ΔCCS% < 2). The separation in the drift time dimension of critical pairs of isomers for modified mycotoxins was also achieved. In addition, the comparison of measured and predicted CCS values, including regulated and emerging mycotoxins, was addressed.

## Introduction

Over the past decade, the hyphenation of ion mobility spectrometry (IMS) with high-resolution mass spectrometry (HRMS) has risen as a powerful technique for the separation, identification, and structural elucidation of analytes across diverse fields of science. The addition of a new dimension of separation to the common workflow will benefit both targeted and nontargeted analysis. On the one hand, when profiling a target class of analytes, IMS enhances the performance characteristics in terms of sensitivity, peak capacity, and compound identification, reducing the false detections.^1^ On the other hand, IMS-MS expands the analyte coverage and increases confidence in the metabolite annotation, which represents the bottleneck of untargeted omics.^2−4^

This is possible because IMS-MS allows the determination of the collision cross section (CCS) that is considered as a structural property of ionized molecules. As a result of these advantages, several research groups have used IMS-MS to build CCS libraries^1,5−9^ in which the measured values serve as additional molecular descriptors for assigning identities to unknown analytes or gain more confidence in the identification of known molecules.

The implementation of IMS within the food analytical field is quite new, and its applicability in routine food safety analysis has been slowed down by the lack of CCS databases for contaminants and residues.

Very recently, a few contaminant databases have been proposed (e.g., mycotoxins, pesticides, veterinary drugs environmental contaminants),^1,6,7^ but they are far away from covering the varying range of contaminants present in food samples.

CCS has been demonstrated to be a good molecular descriptor, being independent from the concentration and the complexity of the matrix^1,4^ and highly reproducible in inter- and intraday studies (variation <1%).^6,8^ There is a consensus that the precision of drift time measurements and with these CCS databases is relatively high; thus, these values can certainly be used with an in-house database.^6,8,10^ There is also evidence that CCS reproducibility is within the range of ±2% (which is normally considered the acceptable error) between identical instruments across different laboratories equipped with traveling wave (TWIMS)^8,11^ and drift tube (DTIMS).^10^ Based on the high reproducibility reported across DTIMS instruments (RSD 0.29%)^10^ and TWIMS (RSD < 1%),^6,8^ some authors proposed to narrow the tolerance threshold to ±1.5% when a same instrument is used.

However, the challenge is to demonstrate whether common CCS databases can be used independently from the instrument type and IMS technologies, which utilize different methodologies for determining the gas-phase mobility. DTIMS relies on the fundamental ion mobility relationship that directly correlates the measured arrival time of an ion to the CCS,^12,13^ whereas in the case of other IM technologies (i.e., TWIMS, ion trapping (TIMS), and structures for loss-less ion manipulation (SLIM)), the CCS value is obtained indirectly by the use of a calibration equation^12,13^ based on universally accepted DTIMS-derived CCS as the reference value.^13^

So far, few studies have investigated the comparability of the CCS determined by different platforms, and the comparison of DTIMS with non-DTIMS still poses the greatest challenge when attempting to use a common database. This is an emerging issue, and an in-depth discussion around the proposal of using CCS information obtained from different IM technologies is ongoing and reported by the ion mobility community.^12^

One of the most comprehensive studies^14^ reported deviations lower than ±1% for most of the considered analytes when comparing CCS obtained using DTIMS and TWIMS. However, some compounds showed deviations of up to 6.2%, which drove the authors to the conclusion that CCS databases cannot be used without care independent of the instrument type. Although more data would be needed, while creating a database, it is good practice to clearly indicate the instrument type used for the CCS determination.

Furthermore, while building a traveling wave CCS (^TW^CCS) database, the calibrant mixture used should also be indicated, the CCS being derived through calibration equation and not directly measured. There is currently no consensus regarding the CCS calibration procedure or the type of calibration compounds to be used.^12^ Originally, TWIM calibration was based on poly-dl-alanine (mass range: 151.1–1154.6 Da; CCS: 130.4–333.6 Å^2^), which was then implemented by the addition of a number of small molecules, which include perfluorinated compounds in the range *m*/*z* 1000–2000 and organic acids for a more comprehensive coverage at low masses in the negative ion mode (Major Mix IMS/time-of-flight (TOF) Calibration Kit—mass range: 151.1–1966.9 Da; CCS: 130.4–372.6 Å^2^). Some research groups build their own calibration mixtures or complement the Major Mix with the analytes of interest.^8^ However, by doing so a further bias is introduced.

Recently, Hernandez-Mesa et al.,^6^ reported a TWIMS interplatform study, demonstrating deviation within the range of ±1.5% between Synapt and Vion for most of the CCS measurement for steroids, while using the same calibration mixture. However, some compounds showed deviations greater than this threshold. In light of these findings, the authors suggested for targeted-screening purposes, the use of a score system in which CCS will have a weight on the final score for peak annotation, depending on the CCS bias ranges, together with the other molecular descriptor, named retention time, accurate mass, and fragmentation pattern. The application of a score system would reduce the risk of discarding a good candidate only based on a CCS deviation threshold.

We recently reported the first ^TW^CCS_N2_ database for mycotoxins, showing its applicability and utility in screening of mycotoxins in real food samples.^7^ The present study aims to extend our previous investigation by evaluating the reproducibility of CCS measurements in an interlaboratory study and across different TWIM-MS systems commercially available. The separation in the drift time dimension of critical pairs of isomers for masked mycotoxins is addressed. In addition, the comparison of measured and predicted CCS values for 53 compounds, including regulated and emerging mycotoxins, will be discussed.

## Material and Methods

### Chemicals and Reagents

LC–MS-grade methanol and LC–MS grade water were purchased from Honeywell (Riedel-de Haen, Germany). Acetic acid 99.99% (Sigma-Aldrich, Germany) and ammonium acetate (Fischer Chemicals, UK) were used as mobile phase modifiers. Leucine-enkephalin [186006013] used as lock mass solution and Major Mix IMS/TOF Calibration Kit [186008113] for mass and CCS calibration were purchased from Waters (Manchester, UK).

A total of 53 analytical standards of mycotoxins were purchased from different manufacturers including Sigma-Aldrich (Taufkirchen, Germany) and Biopure (Tulln, Austria). Zearalenone-14-glucoside (ZEN14Glc) was chemically synthesized and purified in our laboratory. T-2 toxin glucosides were kindly provided by Dr. Susan P. McCormick (National Center for Agricultural Utilization Research, U.S. Department of Agriculture, Peoria, United States). Standards of partially hydrolyzed (pHFB) and hydrolyzed (HFB) fumonisins were prepared by alkaline hydrolysis of FB standard solutions. Further details on the synthesis of these mycotoxins are reported in Note 1, Supporting Information. Mixtures containing different standards were prepared in acetonitrile or methanol, depending on their chemical stability, at a concentration of 2 mg/L and stored in glass vials at −20 °C.

From the stock solutions, three different solutions were prepared (1, 10, 100 μg/L) and diluted in an appropriate solvent, matching the initial conditions of the liquid chromatography (LC) gradient.

### UPLC-IMS-MS Analysis

^TW^CCS_N2_ values were determined employing three commercial TWIM-MS instruments: two Vion IMS quadrupole time-of-flight (QTOF) (resolution ∼20 Ω/ΔΩ fwhm) located in two different laboratories and one Synapt G2-Si (resolution ∼40 Ω/ΔΩ fwhm). UPLC was coupled to each MS system for chromatographic separation prior to ionization. The IMS-MS systems consist of hybrids quadrupole orthogonal acceleration time-of-flight mass spectrometers, in which a stacked ring ion guide, that is, the mobility cell, is positioned before the quadrupole mass filter (Vion configuration) or after the quadrupole and between trap and transfer regions (Synapt configuration). Campuzano and Giles have discussed the evolution of TWIMS technology and the differences between these two TWIMS platforms in detail.^15^ Furthermore, the CCS calibration procedure for the TWIMS technology has been reported^16^ and briefly summarized in the Supporting Information (Note 2).

Nitrogen was used as buffer gas in the three instruments.

#### Vion UK (Vion #1)

The instrument was located at Waters Corporation, Wilmslow, Cheshire, UK. Mycotoxin standard mixes prepared at different concentration levels (1, 10, 100 μg/L) were injected in triplicate, thus obtaining the ^TW^CCS_N2_ from the average of *n* = 9, *n* = 6, or *n* = 3 values, depending on the differences in ionization efficiency.

Data were acquired on an ACQUITY UPLC I-Class system coupled to an ion mobility mass spectrometer Vion IMS QTOF operating in the electrospray mode (ESI^+/–^).

For the chromatographic separation, a reverse-phase C18 BEH column (Waters, UK) with 2.1 × 100 mm and a particle size of 1.7 μm, heated at 35 °C was used. LC solvents were 1 mM ammonium acetate in water (solvent A) and methanol (solvent B), both acidified with 0.5% acetic acid. Initial conditions (0.0–0.5 min) were set to 10% solvent B increased to 90% B in 3 min followed by 1 min at 90% B. Reconditioning was achieved by 1.10 min using initial conditions. The total run time was 6 min.

The mass spectrometry detection was conducted in both positive and negative electrospray ionization modes in the mass range of *m*/*z* 50–1000 under the following source conditions: capillary voltage 0.5 kV for positive and 0.5 kV for negative ion modes, cone voltage 50 V, source temperature 150 °C, desolvation temperature 450 °C, desolvation gas flow 600 L/h. Nitrogen was used as the collision gas. Two independent scans with different collision energies (CE) were alternatively acquired during the run (HDMS^E^ acquisition mode): a low-energy scan (CE 6 eV) to monitor the protonated/deprotonated molecules and other potential adducts, while a high-energy scan (CE ramp 28–42 eV) to fragment the ions traveling through the collision cell.

The TOF analyzer was operated in the sensitivity mode with the following settings: IMS gas (nitrogen) flow rate 25 mL/min, wave velocity 250 m/s, IMS pulse height 45 V. The acquisition rate was 10 Hz. Data acquisition and analysis were performed using UNIFI software (Waters, UK).

#### Vion Spain (Vion #2)

The instrument was located at the Research Institute for Pesticides and Water, University Jaume I, Castellón, Spain. The mycotoxin standards were diluted to different concentrations (1, 10, 100 μg/L) and 5 μL were injected, in triplicates per standard, on a CORTECS C18 2.1 × 100 mm, 2.7 μm fused core column (Waters) kept at 40 °C. Obtained CCS values were averaged over the replicates detected (*n* = 9, 6, or 3).

Data were acquired on an ACQUITY UPLC I-Class system coupled to an ion mobility mass spectrometer Vion IMS QTOF, (Waters, UK) in the electrospray mode (ESI^+/–^).

LC solvents were 0.01% formic acid in water (solvent A) and methanol (solvent B) acidified with 0.01% formic acid. Initial conditions (0.0 min) were set to 10% solvent B increased to 90% B in 14 min, followed by 2 min at 90% B. Reconditioning was achieved by 2.0 min using initial conditions. The total run time was 18 min with a flow rate of 0.3 mL/min.

The mass spectrometry detection was conducted in the electrospray mode in the mass range of *m*/*z* 50–1000. Collision energy ramp 28–56 eV (Vion IMS QTOF, fitted with nitrogen as collision gas). The capillary voltage was 0.7 kV for the positive ESI mode and 2.5 kV for the negative ESI mode. The cone voltage was set at 40 V, the source temperature kept at 120 °C, the desolvation gas at 550 °C with a flow of 1000 L/h.

The TOF analyzer was operated in the sensitivity mode with the following settings: IMS gas (nitrogen) flow rate 25 mL/min, wave velocity 250 m/s, IMS pulse height 45 V. The acquisition rate was 10 Hz. Data acquisition and analysis were performed using UNIFI software (Waters, UK).

#### Synapt UK (Synapt #3)

The instrument was located at Waters Corporation, Wilmslow, Cheshire, UK.

Triplicate injections were performed for each mycotoxin standard mix (100 μg/L). The chromatographic separation was achieved on an ACQUITY UPLC I-Class system with an FTN sample manager. A reverse-phase C_18_ BEH column (Waters) with 2.1 × 100 mm and particle size of 1.7 μm, heated at 35 °C was used. The injection volume was 10 μL, and the flow rate was 0.4 mL/min. LC solvents were 1 mM ammonium acetate in water (aqueous mobile phase, A) and methanol (organic mobile phase, B) both acidified with 0.5% acetic acid. A binary gradient method was used as follows: 3–40% B in 4 min with no initial isocratic holding time, 40–90% B in 6 min, hold for 2 min at 90% B, re-equilibration at 3% B for 3 min prior to next injection. The total run time was 15 min.

The chromatographic system was interfaced with a Synapt G2-S*i* operating in the electrospray mode (ESI^+/–^). The capillary voltage was set to +2.5 and −1.5 kV; the sampling cone voltage was 30 V for both polarities, the cone gas flow 50 mL/min, and the source temperature 150 °C. Desolvation gas temperature was 550 °C with a flow rate of 1000 L/h. Prior to use, the ion mobility cell settings were standardized for by setting the following values: 2 mL/min gas flow for the trap cell, 180 mL/min for the helium cell, and 90 mL/min nitrogen flow in the mobility cell, giving an IM cell pressure of ∼3.2 mBar. The IM wave velocity linearly ramped from 1000 to 300 m/s with a constant pulse height of 40 V. Data were acquired over the mass range of *m*/*z* 50–1200 at 10 spectra per second in the data-independent HDMS^E^ mode, whereby after the separated precursor ions exit the IM cell, they are fragmented in one scan function and transmitted intact in another. Low-energy spectra were acquired at CE 3 eV, while high-energy spectra were acquired with a ramp of the transfer CE from 20 to 35 eV. Argon was used as the collision gas. Mass and CCS calibration was performed with Major Mix, using the same reference points as for Vion. Prior to CCS calibration, the system was switched to the mobility mode and left to equilibrate for 1 h. Leucine-enkephalin was employed as the LockSpray solution at a concentration of 200 pg/μL (infusion rate 10 μL/min) acquired every 30 s to provide a real-time single-point mass and CCS calibration. The instrument was controlled with MassLynx v. 4.2 SCN 983. Raw data were processed on UNIFI software v. 1.9.4.

### Statistical Analysis

All statistical analyses were performed using GraphPad Prism (version 8.4.2, GraphPad Software San Diego, CA). Data correlation was evaluated by Pearson’s correlation test (α = 0.05).

### Prediction of the Theoretical CCS Values

Theoretical CCS were obtained with two different models trained with machine learning approaches, the one proposed by Zhou et al.^17^ namely AllCCS (http://allccs.zhulab.cn/) and the recently published by Ross et al.,^18^ CCSbase (https://ccsbase.net/). In brief, using a training set of experimentally measured CCS, the software employs a machine-learning algorithm able to predict CCS values for novel structures. To calculate the predicted CCS for [M + H]^+^, [M + Na]^+^, [M + NH_4_]^+^, and [M – H]^−^ adducts, the SMILES string of each mycotoxin was imported to both web interfaces, AllCCS Predictor and CCSbase.

## Results and Discussion

In the present work, we extended our previous investigation^7^ by complementing and validating our mobility-derived ^TW^CCS_N2_ database of mycotoxins. We assessed the reproducibility of CCS measurement by means of an interlaboratory test. Furthermore, because different types of TWIM-MS systems are commercially available, it is necessary to validate the comparability of different instrument types, when CCS databases are used independently from the instrument. For this purpose, CCS values were determined and compared for a total of 53 mycotoxins and different adduct states in both positive ([M + H]^+^, [M + Na]^+^, [M + NH_4_] ^+^, and [M + K]^+^) and negative ionization modes ([M – H]^−^and [M + CH_3_COO]^−^).

### CCS Repeatability and Interlaboratory Reproducibility

At first, the mycotoxins database was built using Vion #1. Mycotoxins standard mix prepared at different concentration levels (1, 10, 100 μg/L) were injected per triplicate; therefore, the ^TW^CCS_N2_ values were average over *n* = 9 values (for some cases 6 or 3 because the lowest levels could not be observed). The ^TW^CCS_N2_ values, average, standard deviation, and relative standard deviation (RSD) are summarized in Table S1. On the total of 225 ^TW^CCS_N2_ values considered for both positive and negative ionization modes, the minimum RSD was 0.018%, the average RSD was 0.14% (±0.079%), and the maximum RSD was 0.61%. The majority of ions were within the strictest range of highly reproducible measurements (see Figure S1) recently published by Stow et al.^10^ Indeed, 97% of measurements reported an RSD <0.3%. The high precision of the measured ^TW^CCS_N2_ led us to confidently state that these values can certainly be used with an in-house database for mycotoxin screening.

The ^TW^CCS_N2_ obtained with Vion #1 were then compared with those experimentally derived in a second laboratory (Vion #2). Overall, 100 compounds were detected by both instruments at both sites, with a further detection of only 125 ions either by the first or by the second site. Such differences are not unexpected, given that differences in ionization efficiency between different instruments are frequently observed as reported in previous interlaboratory validation studies.^6,8^ Also, stability issues during transportation of standard mixtures across laboratories should be considered as a source of differences in the compounds detected.

Results from the two Vion instruments demonstrated high precision for the ^TW^CCS_N2_ measurements, showing an overall average interlaboratory RSD of 0.25 ± 0.17% for instruments located in two different laboratories.

**Figure 1:**
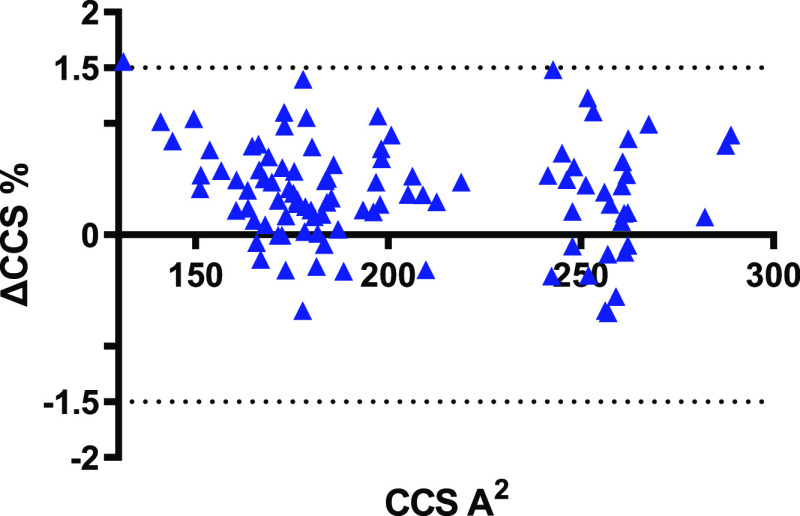
Bland–Altman plot displaying the spread of ^TW^CCS_N2_ percent deviation (ΔCCS%) of values taken from replicate experimental acquisitions on two Vion TWIM-MS instruments located in two different laboratories.

The percentage deviation (ΔCCS%) between the two instruments was calculated keeping the Vion #1 as “reference”. All the ^TW^CCS_N2_ values for the ions detected by Vion#1 and Vion #2 were within the currently accepted error threshold of ±2.0%. In particular, deviations were observed within the range of ±1.5 for 100% of the measurements, within a high percentage of measurements (93%) showing a bias within the range of ±1%, as represented in Figure 1.

Based on these results, when using the same TWIMS instrument type (including the same calibration standards), a threshold of ±1.5% can be considered without assuming a high risk of false negatives when applying cross-laboratory ^TW^CCS_N2_. Narrowing the acceptance error window below 2% in screening analysis will allow higher precision to be achieved in the annotation of molecular candidates. This outcome is in agreement with the result reported recently^6,8^ on the ^TW^CCS_N2_ reproducibility across different laboratories.

### Interplatform CCS Reproducibility

After demonstrating repeatability and reproducibility of the ^TW^CCS_N2_ when the same instrument type is used, we carried out further studies to understand whether a common mycotoxin database can be used independently from the instrument type. To this purpose, the mycotoxin standard mixes were analyzed using a Synapt G2-Si. Overall, 139 common ions were detected by both instrument types (Vion and Synapt) and compared in terms of bias against the database. A graphical comparison of the CCS means for single laboratory (Vion #1, Vion #2, and Synapt G2-Si) is reported in Figure S2.

Synapt G2-Si platform showed high precision, in accordance with the performance of both Vion and Synapt instruments. The average RSD of triplicate measurements was 0.113 ± 0.11%, the minimum RSD was 0.006%, while the maximum RSD was 0.70%. Bar charts displaying the spread of relative standard deviation for both instrument types are depicted in Figure S1.

**Figure 2:**
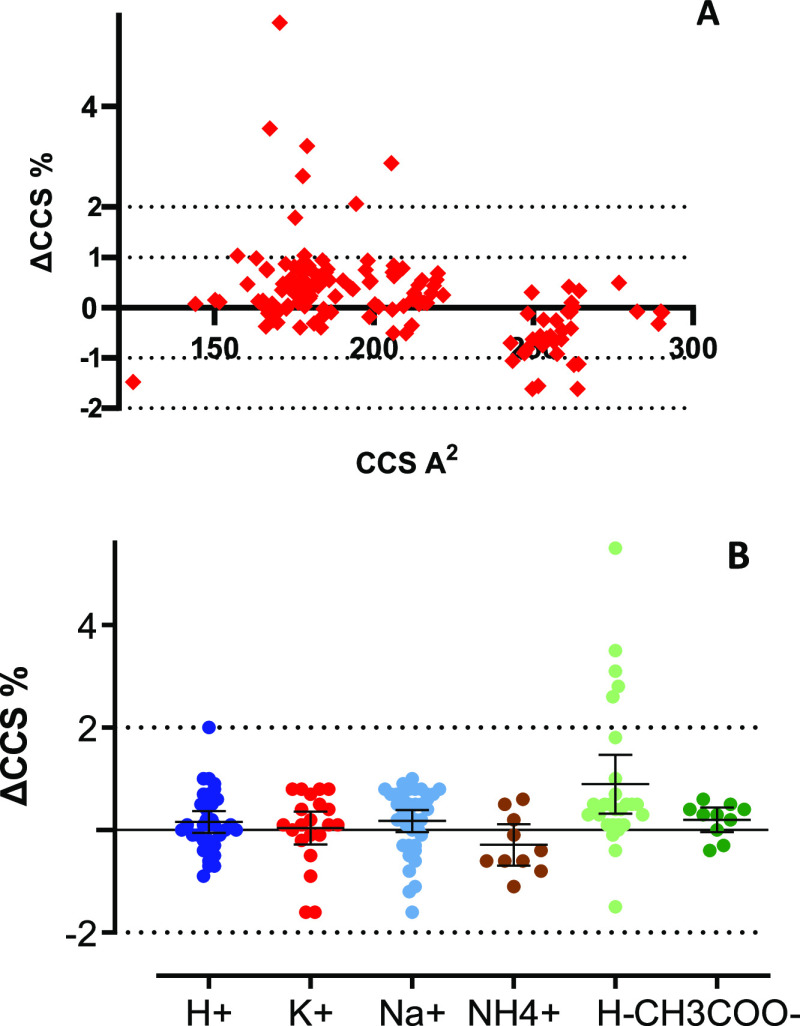
^TW^CCS_N2_ percent deviation of values taken from two different TWIM-MS instruments (Vion vs Synapt) located in two different laboratories. (A) Bland–Altman plot displaying the spread of ^TW^CCS_N2_ percent deviations and (B) their trend according to the adduct ions monitored.

When evaluating the bias between the two T-Wave systems, different performance in terms of reproducibility were found for positive and negative ionization modes. In general, 96.4% of the ^TW^CCS_N2_ measurements were within the error threshold of ±2.0% and interestingly, 89.2% of the ions were within the narrowed error threshold of ±1.0% (see Figure 2A). Very few compounds (*n* = 5) showed deviations greater than the threshold of ±2.0%. The highest deviations were observed for the deprotonated ion of nivalenol (ΔCCS% = 5.5%). The other ions reporting error % higher than ±2.0% were the deprotonated deoxynivalenol (DON) (ΔCCS% = 3.5%), 3-acetyl-DON (ΔCCS% = 3.6%), DON-3-glucoside (ΔCCS% = 2.8), and fusarenon X (ΔCCS% = 3.1%).

Indeed, by further elaborating the data, a trend according to the adduct monitored and the mycotoxin chemical classes was observed (Figure 2B). The highest deviations from the database were observed for the [M – H]^−^ adduct of the type B trichothecene class. These compounds are sesquiterpene epoxides, characterized by multiple protonation and deprotonation sites. Therefore, differences in the CCS might be expected considering the formation of charged isomers depending on the loss of a proton from different molecule sites.

Further investigations are needed to confirm this hypothesis, including the use of high-resolution IMS with improved resolving power, such as cyclic-IMS.

Finally, the database generated within this study was compared with the previously published ^TW^CCS_N2_ data^7^ which were derived from arrival time measurements using a previous generation traveling wave IM-MS instrument, the Synapt HDMS Q-TOF mass spectrometer (from Waters Corporation). It is important to note that the original database obtained from the previous generation TWIM system was created using a different calibrant (i.e. poly-dl-alanine mix, monitoring [M-H_2_O] ions) compared to the calibrant employed in the present work (Major Mix, containing poly-dl-alanine, Ultramark 1621, low-MW acids, and nine additional small molecules, commonly used as QC standards). The exact composition of the different calibration solutions is reported in Tables S3 and S4. Moreover, the first-generation TWIMS technology included different informatics analysis tool, comprising an older peak detection algorithm. Because of the different calibration profiles, slightly higher deviations are to be expected; however, the reported values were still found to be within the common error distribution range. Indeed, for 84.2% of the measurements, deviations were within the threshold of ±2.0%, while the higher errors were found for trichothecenes and aflatoxins monitored as potassium, sodium, and ammonium adducts.

These findings showed that the choice of the calibrants can have an impact, as already discussed elsewhere^12^ but not as high as it might be expected. A systematic error on CCS measurements can be attributed to the intrinsic difference of chemical structure between poly-alanine (linear conformation) and the diversified groups of mycotoxins, which in many cases, share a cyclic-base structure (e.g., trichothecenes, zearalenone and its derivatives, enniatins).

The results presented in this study empirically confirmed the recommendations for reporting ion mobility mass spectrometry measurements recently published,^12^ which suggest that when building a ^TW^CCS database, the calibration mixture used should also be indicated, the CCS being derived through a calibration equation and not directly measured.

### Mycotoxin Isomer Separation

Several isomers have been included in the database, mainly modified mycotoxins, including positional isomers (3- or 15-Ac-DON) or conformational isomers (T-2 α/β-glucoside).

**Figure 3:**
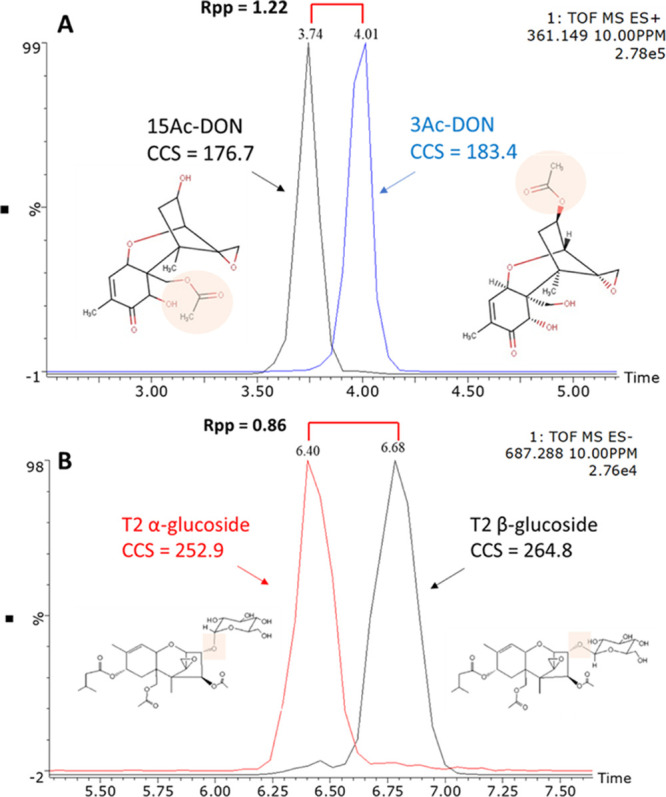
Arrival time distribution (ms) of (A) acetylated forms of DON [M + Na]^+^ and (B) T2 α/β glucoside [M + CH_3_COO]^−^ obtained using the Synapt G2-Si. Rpp: two-peak resolution.

In particular, the drift time separation of acetylated derivatives of DON was investigated considering the challenge of their chromatographic separation. 3- and 15-Ac-DON were detected as protonated, potassium, sodium, and ammonium adducts in the positive and as deprotonated and acetate adducts in the negative mode. Only the sodiated and potassiated species resulted in ^TW^CCS_N2_ values that are significantly different and whose percentage difference is >±2%. Figure 3A shows the separation of the [M + Na]^+^ adduct at *m*/*z* 361.1258 for 3-Ac-DON (CCS 183.4 Å^2^ and 4.01 ms arrival time) and 15-Ac-DON (CCS 176.7 Å^2^ and 3.74 ms arrival time), suggesting a different shape of the ions, which is intensified by the coordination of a sodium atom within the molecular structure.

The separation efficiency was calculated in terms of two-peak resolution (*R*_pp_) using the equation from Dodd et al.,^19^ resulting in a *R*_pp_ > 1 (1.22) and thus indicating that 3- and 15-Ac-DON isomers are resolved in the drift time dimension when the sodium adduct is considered.

Even more challenging is the separation of conformational isomers, as for the different configuration of the anomeric carbon in the T-2 α- and β-glucoside. In this case, the drift time separation was achieved in the negative ionization mode by monitoring the acetate adduct, as shown in Figure 3B. The resolution between the two peaks was *R*_pp_ < 1 (0.86) mainly because of a distortion of the peak at half height (not Gaussian); thus, the two isomers cannot be considered fully resolved, even though the valley is <10% of the peak height.

However, because their CCS percent difference is higher than ±2% (ΔCCS% = 4.3%), T-2 α- and β-glucoside will not be aligned in the drift time dimension, and they will be processed as two different ions. The separation of isomer aids with an increased confidence in the identification process when screening for real samples.

The separation of additional pairs of mycotoxin isomers was further investigated. Although a broad and splitting peak shape was observed, the resolving power of the employed technique was not sufficient to resolve the positional (i.e. zearalenone 14/16 glucoside) and conformational isomers (i.e. α/β zearalenol) analyzed herein. With the improvements in the IMS technology and enhanced resolving power of cyclic IMS, their separation could potentially be possible.

### CCS Prediction

The experimentally derived ^TW^CCS_N2_ can also be compared with the theoretical values, allowing a higher degree of confidence in the identification process. New mycotoxins and modified forms may be discovered and characterized by matching theoretical and experimental rotationally averaged cross-sectional areas, despite the lack of analytical standards.

Theoretical CCS values can be obtained via computational chemistry tools such as MOBCAL,^17,20^ as it was more recently developed by machine learning-based mathematical methods to predict drift times or CCS.^20^

Here, the theoretical CCS was predicted using machine learning based on AllCCS^17^ and CCSbase^18^ online tools. Overall, 155 and 189 ions were considered for AllCCS and CCSbase, respectively. The difference is due to the prediction of potassium adducts that was not available in AllCCS. Predicted CCS values were found to be highly correlated (Pearson *r* > 0.98, see Supporting Information) with the experimentally observed values (^TW^CCS_N2_—Vion#1), as depicted in Figure S3. Despite the power of artificial intelligence, high deviations were found, with prediction errors within ±2% only for 39% of the analytes, while 91% of the compounds fell in the range ±5% of percentage difference when the AllCCS prediction model is considered. Interestingly, greater deviations were found for the protonated adducts when compared with sodium, ammonium, and potassium adducts (see Figure S3).

A possible explanation of the bias observed could be that the CCS data used to build the training set were indeed ^DT^CCS_N2_ using the stepped field method.^17^ To test the real suitability of the model algorithm for the prediction of TW-derived CCS, a training set composed by ^TW^CCS_N2_ would be needed.

On the other hand, the CCSbase^18^ prediction model provides much more comprehensive coverage of structures that also include measurements on TWIM platforms. Indeed, lower deviations were found, with half of the analytes (50.3%) displaying prediction errors within ±2% (see Figure S3 and Table S5).

Because a percentage deviation >5% would not be acceptable because of unlikely applicability, the results obtained in the present study confirmed that prediction models are not completely universal for small molecules.^21^ At least for mycotoxins, building a theoretical CCS database is not reliable when using machine-learning approaches based on a training model that was not constructed with the same class of chemical compounds and experimentally derived using the same IMS technology.

However, research is ongoing, and preliminary data are highly encouraging.^21^ This highlights the importance of creating and validating reliable databases, which ultimately can aid with improved validation of predicted CCS for natural toxins and for other food contaminants. The final, and perhaps holistic goal being the ability to predict the CCS of compounds, for which standards are not readily available, thus bringing about great benefit for future applications in food safety.

In conclusion, the mycotoxin CCS database can be used independently for TWIMS instruments (Vion and Synapt), because 96.4% of the ^TW^CCS_N2_ measurements, were within the error threshold of ±2.0%. The remaining 4% was because of a specific class of mycotoxins, and further studies are already ongoing to investigate the presence of eventual charge isomers, whose effect is impactful in the measurement of CCS for deprotonated species.

Regarding the theoretical CCS, even though results collected so far are highly promising, we are far from relying on predicted CCS values, and further studies are required before proposing the use of CCS as a molecular parameter as such that can be universally applied on all commercial IM-MS platforms. On the other hand, the implementation of a score system based on different ranges of bias between CCS measurements and values in databases seems to be a preferable approach which does not compromise the validity of the databases developed so far.

## Supporting Information Available

Chemical synthesis of zearalenone-14-glucoside and hydrolyzed fumonisins, CCS Calibration of TWIMS-MS, bar charts displaying the spread of relative standard deviation (%) of CCS values taken from replicate experimental acquisitions on Vion IMS QTof and Synapt G2-Si instruments, representation of the ^TW^CCS_N2_ values (Å^2^) measured for each ion by the three TWIMS instruments, CCS-based prediction values versus observed ^TW^CCS_N2_ obtained with AllCCS (A) and CCSbase (C) online tools, mycotoxin database built using TWIM-MS Vion IMS QTof, nitrogen as buffer gas, and Major Mix IMS/TOF as calibrants, mycotoxin database built using TWIM-MS Synapt G2-Si, nitrogen as buffer gas, and Major Mix IMS/TOF as calibrants, composition of CCS calibration solution used for the positive ion mode, composition of CCS calibration solution used for the negative ion mode, theoretical CCS (Å^2^) for mycotoxins obtained using AllCCS and CCSbase prediction models (PDF)

## Abbreviations Used

CCS: collision cross section
IM-MS: ion mobility mass spectrometry
RSD: relative standard deviation
HRMS: high-resolution mass spectrometry
TWIMS: traveling wave ion mobility spectrometry
DTIMS: drift tube ion mobility spectrometry
^TW^CCS_N2_: collision cross section derived using traveling wave ion mobility spectrometry and nitrogen as buffer gas
DON: deoxynivalenol
3- or 15-Ac-DON: acetyl-deoxynivalenol
UPLC: ultra performance liquid chromatography
