# Supplementary material for: Travelling Wave Ion Mobility-Derived Collision Cross Section for Mycotoxins: Investigating Interlaboratory and Interplatform Reproducibility
Source: J Agric Food Chem. 2020 Sep 1;68(39):10937–43. doi: 10.1021/acs.jafc.0c04498 (PMC8154562; doi:10.1021/acs.jafc.0c04498)
Supplement: Supplementary file 1 — jf0c04498_si_001.pdf [file jf0c04498_si_001.pdf]

Supporting Information for:

Travelling Wave Ion Mobility-Derived Collision Cross Section for Mycotoxins: investigating interlaboratory and interplatform reproducibility

Laura Righetti[1*], Nicola Dreolin[2], Alberto Celma[3], Mike McCullagh[2], Gitte Barknowitz[2], Juan V. Sancho[3], Chiara Dall'Asta[1]

[1] Department of Food and Drug, University of Parma, Viale delle Scienze 17/A, I-43124 Parma, Italy

[2] Waters Corporation, Altrincham Road, SK9 4AX Wilmslow, United Kingdom

[3] Environmental and Public Health Analytical Chemistry, Research Institute for Pesticides and Water, University Jaume I, Avda. Sos Baynat s/n, E-12071 Castellón, Spain

***Corresponding authors' detail:**

Dr. Laura Righetti, Department of Food and Drug, University of Parma, Viale delle Scienze 17/A, I-43124 Parma, Italy.

E-mail laura.righetti@unipr.it, phone contact +39 0521 906196

Note 1 - Chemical synthesis of zearalenone-14-glucoside and hydrolysed fumonisins

- Zearalenone-14-glucoside (ZEN14Glc) was chemically synthesized and purified in our laboratory, according to Zill et al.1990[1], with slight modifications. Zearalenone (25 mg) was dissolved in chloroform and added to acetobromoglucose (2,3,4,6- tetra-O-acetyl-α-D-glucopyranosylbromide, 1 g) and tetrabutylammonium bromide (252 mg) as transfer phase catalyst (molar ratio 1:30:10), both dissolved in 50 mM Cs_2CO_3. The mixture was magnetically stirred for 24 h at 30 °C. Afterward, the organic layer was collected; the aqueous layer was washed with chloroform, and the organic layers were pooled and reduced to dryness under vacuum. The residue was re-dissolved in 0.1 N NaOH and stirred for 5 h to allow ZEN14Glc deprotection. After neutralization with CH_3COOH, the final product was evaporated to dryness and the residue dissolved in methanol and stored at −8 °C. The reaction yield was calculated as 88%. The reaction was checked by UHPLC-HRMS and purified using semipreparative LC-UV system. The final product was characterized by 1H- and 13C-NMR after dissolving 1 mg in CD3OD (1 mL).

- Standards of partially hydrolysed (pHFB) and hydrolysed (HFB) fumonisins were prepared by alkaline hydrolysis of FB standard solutions, following the protocol reported by Dall'Asta et al. 2008.[2] A standard solution of the three main fumonisins (50 g/mL of each, 5 mL) was prepared in ACN/water 1:1 and evaporated to dryness. The residue was dissolved in 2 M KOH (5 mL), then allowed to react over-night at room temperature. After hydrolysis, the mixture was extracted three times by liquid–liquid partition using ACN (5 mL each aliquot). The organic phases were pooled, evaporated under nitrogen stream and re-dissolved in 1 mL of methanol. The reaction yield was checked by LC-MS, by monitoring the conversion of FB1 to HFB1 and the absence of side products, and it was found to be higher than 99%.

References

1. Zill, G., Ziegler, W., Engelhardt, G., and Wallnöfer, P. R. (1990) Chemically and biologically synthesized zearalenone-4-β-D-glucopyranoside: Comparison and convenient determination by gradient HPLC. Chemosphere 21, 435−442.
2. Dall'Asta C, Galaverna G, Mangia M, Sforza S, Dossena A, Marchelli R. 2009. Free and bound fumonisins in gluten-free food products. Mol Nutr Food Res 53:492–499

Note 2 – CCS Calibration of TWIMS-MS

On the Vion systems the CCS calibration is performed automatically using UNFI software, whilst the CCS calibration on the Synapt G2-Si is automatically performed by IntelliStart software embedded into MassLynx software. In both cases the composition of the calibrant mix (named Major Mix) and the reference CCS values are reported in Table S3 and S4. The calibration function is represented by the following power-law equation:

$$\Omega_{\boldsymbol{n}} = A(t_A - t_0)^B$$

Where $\Omega_{\boldsymbol{n}}$ is the normalized collision cross section, the coefficient A and the exponential factor B are empirically calculated, t_A is the arrival time, and t_0 is the undetermined time offset, intended to capture any additional timing delays in the system, and is assumed constant for all ions.

The collision cross section (Ω) is calculated by the following formula:

$$\Omega = \Omega_n * \frac{z}{\sqrt{\mu}}$$

Where z is the charge of the calibrant, and μ is the reduced mass:

$$\mu = \frac{m_{ion} * m_{gas}}{m_{ion} + m_{gas}}$$

m_{ion} is the mass of the calibrant and m_{gas} is the mass of the buffer gas (N_2).

The calibration function and the percentage CCS deviations are stated for each point (acceptance criteria ≤ ±2.00 ΔCCS%) and are present in the calibration report. The calibration settings are used throughout the analysis.

Detailed description of the calibration procedure in TWIMS has been provided by previous works.

References

1. Righetti L.et al. (2020) Methods Mol Biol. 2084:133-144. doi:10.1007/978-1-0716-0030-6_8
2. Ruotolo B. T. et al. (2008) Nature Protocols, Vol. 3, N. 7, 1139
3. Bush M. et al. (2010) Anal. Chem., 82, 9557–9565
4. Bush M. et al. (2012) Anal. Chem., 84, 16, 7124–7130
5. Campuzano I. et al. (2012) Anal. Chem., 84, 1026-1033

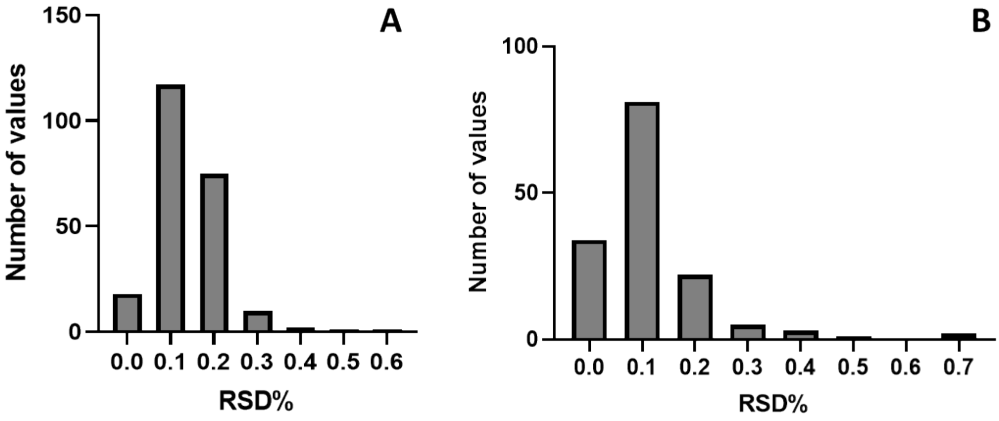

Figure 1S. Bar charts displaying the spread of relative standard deviation (%) of CCS values taken from replicate experimental acquisitions on (A) Vion IMS QTof and (B) Synapt G2-Si instruments.

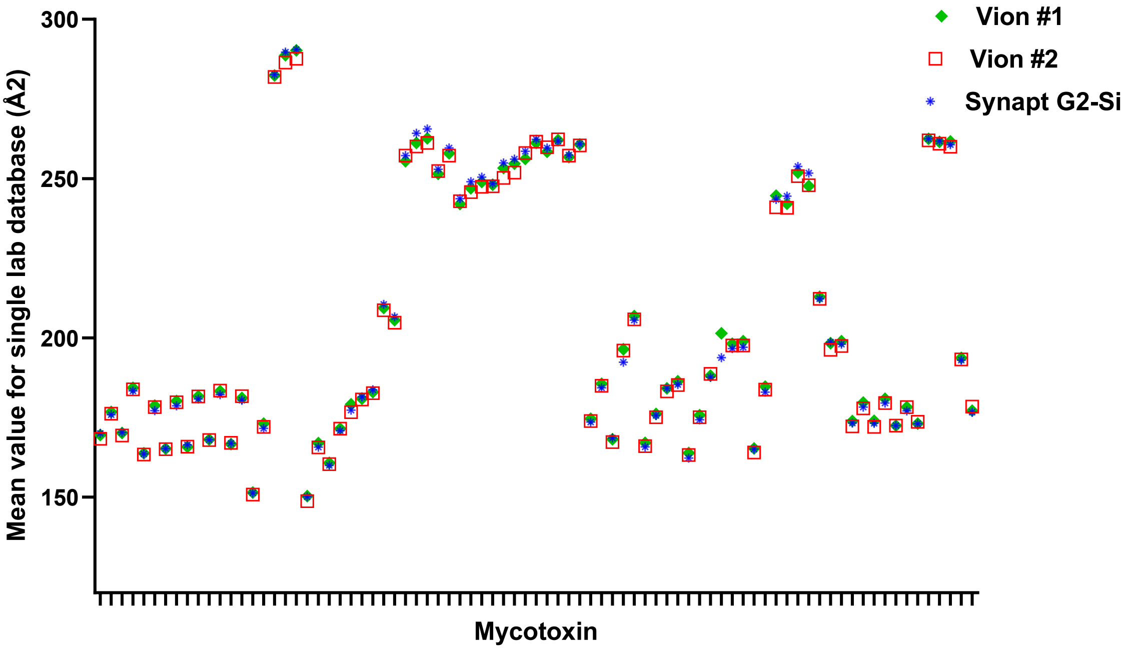

Figure 2S. Representation of the $^{TW}CCS_{N2}$ values (Å^2) measured for each ion by the three TWIMS instruments.

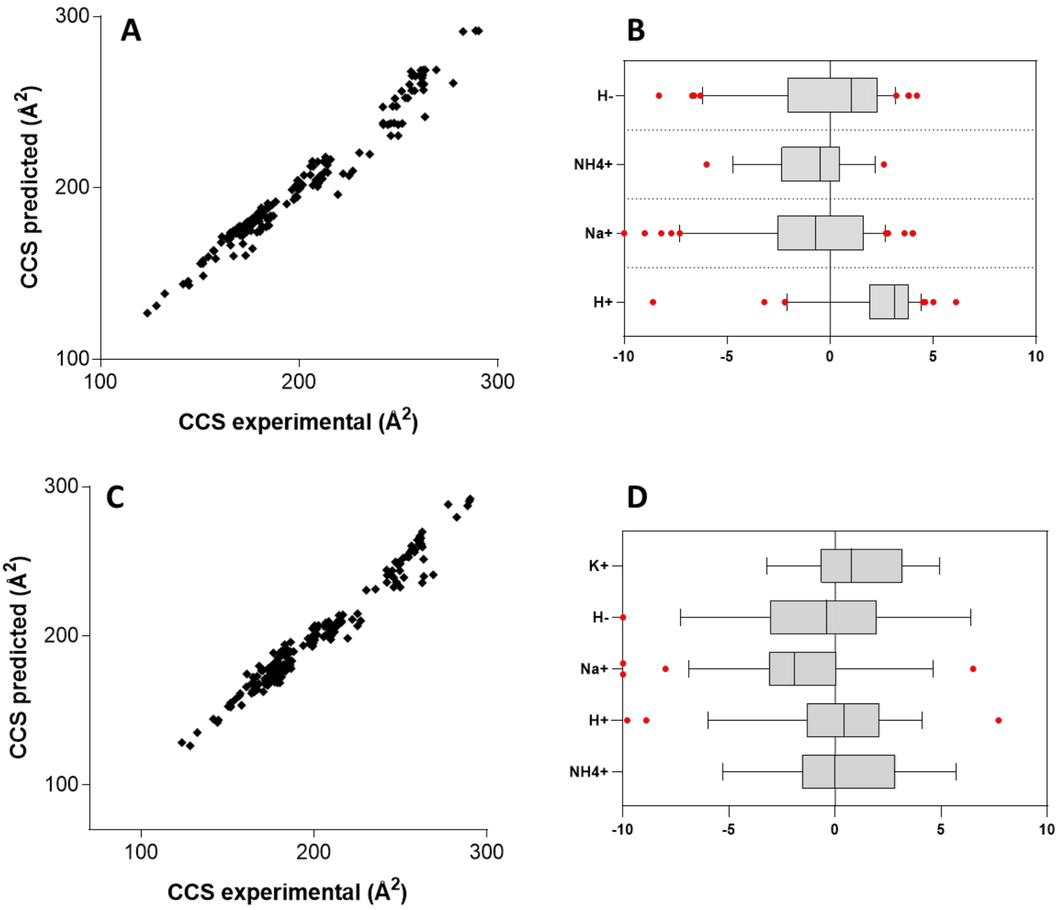

Figure 3S. (A, C) CCS based prediction values vs. observed $^{TW}CCS_{N2}$ obtained with AllCCS (A) and CCSbase (C) on-line tools. Data were found to be highly correlated for both prediction models (A: Pearson r = 0.9838; $p < 0.001$; C: Pearson r = 0.9842; $p < 0.001$) (B, D) Spread of CCS percent deviations (range ΔCCS= ±10%) according to the adduct ions monitored obtained with AllCCS (B) and CCSbase (D) prediction models. Potassium adduct is displayed only in the CCSbase (D) data because it was not available for prediction in AllCCS (B).

Table S1. Mycotoxin database built using TWIM-MS Vion IMS QTof, nitrogen as buffer gas and Major Mix IMS/TOF as calibrants.

Mycotoxin	Adduct	CCS±SD (Å^2)			n*	RSD%
15-Acetyldeoxynivalenol	+CH3COO	185.61	±	0.11	6	0.06
15-Acetyldeoxynivalenol	+H	169.5	±	0.13	6	0.08
15-Acetyldeoxynivalenol	-H	176.47	±	0.23	6	0.13
15-Acetyldeoxynivalenol	+Na	176.75	±	0.24	6	0.14
3-Acetyldeoxynivalenol	+Na	184.37	±	0.14	6	0.08
3-Acetyldeoxynivalenol	+CH3COO	184.32	±	0.18	6	0.10
3-Acetyldeoxynivalenol	+NH4	179.93	±	0.18	6	0.10
3-Acetyldeoxynivalenol	-H	179.99	±	0.22	6	0.12
3-Acetyldeoxynivalenol	+H	170.2	±	0.24	6	0.14
3-Acetyldeoxynivalenol	+K	186.63	±	0.28	6	0.15
Aflatoxin B1	-H	171.94	±	0.11	6	0.06
Aflatoxin B1	+H	163.8	±	0.32	9	0.20
Aflatoxin B1	+NH4	252.39	±	0.52	6	0.21
Aflatoxin B1	+K	177.79	±	0.38	9	0.21
Aflatoxin B1	+Na	178.76	±	0.48	9	0.27
Aflatoxin B2	+NH4	256.82	±	0.2	6	0.08
Aflatoxin B2	+Na	180.19	±	0.17	9	0.09
Aflatoxin B2	+H	165.27	±	0.16	9	0.10
Aflatoxin B2	-H	174.03	±	0.19	9	0.11
Aflatoxin B2	+K	179.39	±	0.25	9	0.14
Aflatoxin G1	+K	180.9	±	0.18	9	0.10
Aflatoxin G1	-H	174.88	±	0.18	9	0.10
Aflatoxin G1	+H	165.78	±	0.24	9	0.14
Aflatoxin G1	+Na	181.68	±	0.28	9	0.15
Aflatoxin G1	+NH4	257.85	±	0.44	6	0.17
Aflatoxin G2	+K	182.88	±	0.15	9	0.08
Aflatoxin G2	+NH4	264.58	±	0.26	6	0.10
Aflatoxin G2	+Na	183.33	±	0.22	9	0.12
Aflatoxin G2	-H	176.89	±	0.3	9	0.17
Aflatoxin G2	+H	168.13	±	0.44	9	0.26
Aflatoxin M1	+K	180.21	±	0.13	9	0.07
Aflatoxin M1	-H	173.13	±	0.13	6	0.08
Aflatoxin M1	+Na	181.18	±	0.15	6	0.08
Aflatoxin M1	+H	166.71	±	0.14	6	0.08
Aflatoxin M1	+NH4	237.75	±	0.21	6	0.09
Alternariol	+H	151.48	±	0.23	6	0.15
Alternariol	-H	151.64	±	0.25	6	0.16
Alternariol	+Na	173.09	±	0.3	6	0.17
Alternariol-methylether	+H	154.3	±	0.18	6	0.12
Alternariol-methylether	-H	156.76	±	0.19	6	0.12
Alternariol-methylether	+Na	176.51	±	0.26	6	0.15
Beauvericin	-H	277.5	±	0.12	6	0.04
Beauvericin	+Na	288.77	±	0.46	6	0.16
Beauvericin	+NH4	290.22	±	0.5	6	0.17
Beauvericin	+CH3COO	293.76	±	0.52	6	0.18

Beauvericin	+K	289.77	±	0.52	5	0.18
Beauvericin	+H	282.37	±	0.62	6	0.22
Citrinin	-H	157.96	±	0.09	6	0.06
Citrinin	+K	164.42	±	0.18	6	0.11
Citrinin	+Na	166.98	±	0.2	6	0.12
Citrinin	+H	150.33	±	0.2	6	0.13
Cyclopiazonic acid	+H	173.65	±	0.13	6	0.07
Cyclopiazonic acid	+Na	187.04	±	0.33	6	0.18
Deoxynivalenol	+CH3COO	175.17	±	0.19	6	0.11
Deoxynivalenol	+Na	171.5	±	0.25	6	0.15
Deoxynivalenol	+H	160.75	±	0.26	6	0.16
Deoxynivalenol	-H	170.25	±	0.32	6	0.19
Deoxynivalenol	+K	173.3	±	0.33	6	0.19
Diacetoxyscirpenol	+K	183.32	±	0.35	6	0.19
Diacetoxyscirpenol	+Na	180.93	±	0.39	6	0.22
Diacetoxyscirpenol	+NH4	182.98	±	0.41	6	0.22
Diacetoxyscirpenol	+H	179.15	±	0.59	6	0.33
DON-3-glucoside	+CH3COO	211.45	±	0.23	6	0.11
DON-3-glucoside	+NH4	210.57	±	0.34	6	0.16
DON-3-glucoside	+K	209.43	±	0.37	6	0.18
DON-3-glucoside	+Na	205.55	±	0.37	6	0.18
DON-3-glucoside	+H	209.65	±	0.41	6	0.20
DON-3-glucoside	-H	208.39	±	0.41	6	0.20
Enniatin A	+NH4	262.63	±	0.45	6	0.17
Enniatin A	+Na	261.22	±	0.52	6	0.20
Enniatin A	+H	255.46	±	0.61	6	0.24
Enniatin A	+K	261.63	±	0.93	6	0.36
Enniatin A1	+Na	256.59	±	0.28	6	0.11
Enniatin A1	+NH4	257.96	±	0.32	6	0.12
Enniatin A1	+H	251.47	±	0.48	6	0.19
Enniatin B	-H	249.79	±	0.32	6	0.13
Enniatin B	+H	242	±	0.38	6	0.16
Enniatin B	+Na	246.96	±	0.47	6	0.19
Enniatin B	+NH4	248.93	±	0.49	6	0.20
Enniatin B1	+Na	253.34	±	0.34	6	0.13
Enniatin B1	+NH4	254.66	±	0.36	6	0.14
Enniatin B1	+H	248.1	±	0.48	9	0.19
Fumonisin B1	-H	262.52	±	0.12	9	0.05
Fumonisin B1	+H	256.23	±	0.22	9	0.09
Fumonisin B1	+Na	261.21	±	0.3	6	0.11
Fumonisin B1	+K	261.25	±	0.57	6	0.22
Fumonisin B2	-H	261.5	±	0.18	9	0.07
Fumonisin B2	+Na	262.06	±	0.21	6	0.08
Fumonisin B2	+H	258.41	±	0.24	9	0.09
Fumonisin B2	+K	259.78	±	0.41	5	0.16
Fumonisin B3	+Na	260.7	±	0.12	6	0.05
Fumonisin B3	+H	256.74	±	0.21	6	0.08
Fumonisin B3	-H	261.71	±	0.22	6	0.08
Fumonisin B3	+K	260.34	±	0.35	3	0.13

Fusarenon X	+CH3COO	186.37	±	0.12	6	0.06
Fusarenon X	+Na	185.6	±	0.18	6	0.10
Fusarenon X	-H	181.86	±	0.22	6	0.12
Fusarenon X	+NH4	183.5	±	0.33	3	0.18
Fusarenon X	+H	174.6	±	0.33	6	0.19
Fusarenon X	+K	186.11	±	0.54	6	0.29
Gliotoxin	+Na	168.18	±	0.3	6	0.18
Gliotoxin	-H	164.67	±	0.36	6	0.22
Gliotoxin	+H	161.08	±	0.68	6	0.42
HT-2 Toxin	+CH3COO	218.37	±	0.16	9	0.07
HT-2 Toxin	+K	209.83	±	0.17	6	0.08
HT-2 Toxin	+Na	206.88	±	0.2	6	0.10
HT-2 Toxin	+H	196.41	±	0.24	6	0.12
HT-2 Toxin	+NH4	209.34	±	0.43	6	0.21
Hydrolized FB1	+CH3COO	221.89	±	0.07	6	0.03
Hydrolized FB1	-H	211.76	±	0.22	6	0.10
Hydrolized FB1	+H	206.64	±	0.49	6	0.24
Hydrolized FB1	+K	216.7	±	0.61	6	0.28
Hydrolized FB1	+Na	213.19	±	0.61	6	0.29
Hydrolized FB2	+CH3COO	219.29	±	0.04	6	0.02
Hydrolized FB2	+H	205.72	±	0.08	6	0.04
Hydrolized FB2	+Na	212.34	±	0.33	6	0.16
Hydrolized FB2	-H	209.72	±	0.37	6	0.18
Hydrolized FB2	+K	215.81	±	0.41	6	0.19
Hydrolized FB3	+CH3COO	220.22	±	0.04	6	0.02
Hydrolized FB3	+H	207.02	±	0.18	6	0.09
Hydrolized FB3	+Na	209.27	±	0.32	6	0.15
Hydrolized FB3	+K	214.26	±	0.39	6	0.18
Hydrolized FB3	-H	208.12	±	0.4	6	0.19
Meleagrin	-H	199.48	±	0.1	6	0.05
Meleagrin	+Na	209.5	±	0.38	6	0.18
Meleagrin	+H	201.67	±	0.46	6	0.23
Mycophenolic acid	-H	176.26	±	0.19	6	0.11
Mycophenolic acid	+Na	176.12	±	0.19	6	0.11
Mycophenolic acid	+K	178.61	±	0.21	6	0.12
Mycophenolic acid	+H	167.03	±	0.2	6	0.12
Neosolaniol	+NH4	186.41	±	0.12	6	0.06
Neosolaniol	+Na	184.15	±	0.12	6	0.07
Neosolaniol	+K	186.43	±	0.13	6	0.07
Neosolaniol	+CH3COO	202.51	±	0.25	6	0.12
Neosolaniol	+H	184.19	±	0.39	6	0.21
Nivalenol	-H	175.34	±	0.1	6	0.06
Nivalenol	+Na	175.72	±	0.15	6	0.09
Nivalenol	+H	163.89	±	0.14	6	0.09
Nivalenol	+CH3COO	178.25	±	0.16	6	0.09
Nivalenol	+NH4	173.47	±	0.19	3	0.11
Nivalenol	+K	177.05	±	0.23	6	0.13
Ochratoxin A	-H	193.67	±	0.11	9	0.06
Ochratoxin A	+Na	198.17	±	0.19	6	0.10

Ochratoxin A	+K	201.42	±	0.22	6	0.11
Ochratoxin A	+H	188.12	±	0.29	6	0.15
Patulin	+H	128.24	±	0.05	3	0.04
Patulin	-H	123.58	±	0.3	6	0.24
Paxilline	-H	214.18	±	0.13	6	0.06
Paxilline	+K	225.12	±	0.31	6	0.14
Paxilline	+Na	226.98	±	0.35	6	0.15
Paxilline	+CH3COO	226.78	±	0.61	6	0.27
Penicillic acid	+Na	144.62	±	0.17	6	0.12
Penicillic acid	+H	132.35	±	0.25	6	0.19
Penitrem A	+Na	263.27	±	0.4	6	0.15
Phomopsin A	+Na	268.93	±	0.08	3	0.03
Phomopsin A	-H	262.51	±	0.12	6	0.05
Phomopsin A	+H	263.42	±	0.24	6	0.09
Roquefortine C	-H	199.12	±	0.27	6	0.14
Roquefortine C	+H	198.94	±	0.27	6	0.14
Stachybotrylactam	-H	200.33	±	0.17	6	0.08
Stachybotrylactam	+H	197.3	±	0.32	6	0.16
Stachybotrylactam	+Na	219.43	±	0.42	6	0.19
Sterigmatocystin	+CH3COO	188.06	±	0.13	3	0.07
Sterigmatocystin	+Na	184.73	±	0.2	6	0.11
Sterigmatocystin	+H	165.33	±	0.2	6	0.12
Sterigmatocystin	+K	184.41	±	0.4	6	0.22
T-2 Glucoside alpha	+CH3COO	252.85	±	0.05	6	0.02
T-2 Glucoside alpha	+NH4	245.98	±	0.11	6	0.04
T-2 Glucoside alpha	+Na	242.13	±	0.13	6	0.05
T-2 Glucoside alpha	+H	244.63	±	0.17	6	0.07
T-2 Glucoside alpha	+K	245.17	±	0.19	6	0.08
T-2 Glucoside alpha	-H	246.07	±	0.26	6	0.11
T-2 Glucoside beta	+CH3COO	264.83	±	0.07	6	0.03
T-2 Glucoside beta	+NH4	251.9	±	0.26	6	0.10
T-2 Glucoside beta	-H	249.78	±	0.3	6	0.12
T-2 Glucoside beta	+K	249.59	±	0.37	6	0.15
T-2 Glucoside beta	+Na	247.66	±	0.41	6	0.17
T-2 Glucoside beta	+H	242.24	±	0.41	6	0.17
T-2 Toxin	+Na	212.91	±	0.08	6	0.04
T-2 Toxin	+K	215.67	±	0.21	6	0.10
T-2 Toxin	+CH3COO	225.49	±	0.22	9	0.10
T-2 Toxin	+H	202.4	±	0.22	6	0.11
T-2 Toxin	+NH4	214.36	±	0.51	6	0.24
Tentoxin	+K	200.33	±	0.2	6	0.10
Tentoxin	-H	199.47	±	0.22	6	0.11
Tentoxin	+Na	199.01	±	0.23	6	0.12
Tentoxin	+H	198.34	±	0.43	6	0.22
Tenuazonic acid	-H	144.11	±	0.29	6	0.20
Tenuazonic acid	+H	141.63	±	0.35	6	0.25
Tenuazonic acid	+Na	151.76	±	0.93	6	0.61
Verrucarol	+K	170.6	±	0.16	4	0.09
Verrucarol	+CH3COO	176.56	±	0.2	3	0.11

Verrucarol	+NH4	165.41	±	0.2	6	0.12
Verrucarol	+Na	171.7	±	0.26	6	0.15
Verrucarol	+H	157.16	±	0.33	6	0.21
Verruculogen	+Na	235.44	±	0.18	6	0.08
Verruculogen	+CH3COO	238.43	±	0.31	6	0.13
Verruculogen	-H	230.22	±	0.32	6	0.14
Zearalenol-alpha	-H	179.28	±	0.1	6	0.06
Zearalenol-alpha	+CH3COO	193.22	±	0.13	6	0.07
Zearalenol-alpha	+H	173.95	±	0.27	6	0.16
Zearalenol-alpha	+Na	179.74	±	0.37	6	0.21
Zearalenol-alpha	+K	182.23	±	0.53	6	0.29
Zearalenol-beta	-H	178.69	±	0.13	6	0.07
Zearalenol-beta	+CH3COO	193.02	±	0.2	6	0.10
Zearalenol-beta	+H	173.99	±	0.39	6	0.22
Zearalenol-beta	+Na	180.95	±	0.51	6	0.28
Zearalenol-beta	+K	182.1	±	0.54	6	0.30
Zearalenone	-H	177.17	±	0.11	9	0.06
Zearalenone	+Na	178.3	±	0.32	6	0.18
Zearalenone	+CH3COO	190.81	±	0.36	6	0.19
Zearalenone	+H	172.47	±	0.35	9	0.20
Zearalenone	+K	178.83	±	0.39	6	0.22
Zearalenone-14-glucoside	+Na	215.78	±	1.12	5	0.52
Zearalenone-14-glucoside	+CH3COO	228.72	±	0.07	6	0.03
Zearalenone-14-glucoside	-H	222.57	±	0.1	6	0.04
Zearalenone-16-glucoside	-H	220.76	±	0.1	6	0.05
Zearalenone-16-glucoside	+CH3COO	228.14	±	0.18	6	0.08

***n = number of detected replicates**

Table S2. Mycotoxin database built using TWIM-MS Synapt G2-Si, nitrogen as buffer gas and Major Mix IMS/Tof as calibrants.

Mycotoxin	Adduct	CCS±SD (Å^2)	RSD%
15-Acetyldeoxynivalenol	+H	169.98 ± 0.70	0.41
15-Acetyldeoxynivalenol	+K	177.72 ± 0.35	0.20
15-Acetyldeoxynivalenol	+Na	175.90 ± 0.28	0.16
15-Acetyldeoxynivalenol	-CH2O - H	167.15 ± 0.25	0.15
3-Acetyldeoxynivalenol	+H	170.28 ± 0.04	0.03
3-Acetyldeoxynivalenol	+Na	183.42 ± 0.03	0.02
3-Acetyldeoxynivalenol	+NH4	179.64 ± 0.31	0.17
3-Acetyldeoxynivalenol	-H	175.36 ± 0.58	0.33
3-Acetyldeoxynivalenol	-CH2O - H	166.50 ± 0.16	0.10
Aflatoxin B1	+H	163.62 ± 0.11	0.07
Aflatoxin B1	+K	176.35 ± 0.13	0.07
Aflatoxin B1	+Na	177.18 ± 0.13	0.07
Aflatoxin B1	-H	171.09 ± 0.02	0.01
Aflatoxin B2	+H	165.17 ± 0.02	0.02
Aflatoxin B2	+K	178.00 ± 0.05	0.03
Aflatoxin B2	+Na	178.75 ± 0.08	0.05
Aflatoxin B2	-H	173.18 ± 0.10	0.06
Aflatoxin G1	+H	166.39 ± 0.12	0.07
Aflatoxin G1	+K	180.20 ± 0.07	0.04
Aflatoxin G1	+Na	180.76 ± 0.15	0.08
Aflatoxin G2	+H	168.00 ± 0.10	0.06
Aflatoxin G2	+K	182.07 ± 0.23	0.12
Aflatoxin G2	+Na	182.23 ± 0.21	0.12
Aflatoxin G2	-H	173.77 ± 0.12	0.07
Aflatoxin M1	+H	166.89 ± 0.08	0.05
Aflatoxin M1	+K	179.81 ± 0.09	0.05
Aflatoxin M1	+Na	180.43 ± 0.09	0.05
Aflatoxin M1	-H	173.03 ± 0.05	0.03
Alternariol	+H	151.32 ± 0.14	0.09
Alternariol	+Na	171.64 ± 0.39	0.22
Alternariol	-H	151.43 ± 0.09	0.06
Beauvericin	+H	282.63 ± 0.08	0.03
Beauvericin	+K	289.99 ± 0.39	0.14
Beauvericin	+Na	289.74 ± 0.48	0.17
Beauvericin	+NH4	290.46 ± 0.07	0.03
Beauvericin	-H	276.14 ± 0.93	0.34
Citrinin	+H	150.14 ± 0.21	0.14
Citrinin	+Na	165.66 ± 0.22	0.13
Citrinin	-H	156.38 ± 0.08	0.05
Deoxynivalenol	+H	159.97 ± 0.08	0.05
Deoxynivalenol	+Na	170.88 ± 0.13	0.08
Deoxynivalenol	-H	164.35 ± 0.19	0.12
Deoxynivalenol	-CH2O - H	158.80 ± 0.08	0.05
Diacetoxyscirpenol	+H	177.31 ± 0.12	0.07
Diacetoxyscirpenol	+K	183.67 ± 0.16	0.09
Diacetoxyscirpenol	+Na	181.48 ± 0.13	0.07
Diacetoxyscirpenol	+NH4	183.73 ± 0.35	0.19

DON-3-glucoside	+K	210.47	±	0.28	0.13
DON-3-glucoside	+Na	206.59	±	0.18	0.09
DON-3-glucoside	-H	202.51	±	0.11	0.06
DON-3-glucoside	-CH2O - H	192.63	±	0.09	0.05
DON-3-glucoside	+CH3COO	212.25	±	0.07	0.03
Enniatin A	+H	257.21	±	0.49	0.19
Enniatin A	+K	265.86	±	0.54	0.20
Enniatin A	+Na	264.23	±	0.49	0.19
Enniatin A	+NH4	265.61	±	0.63	0.24
Enniatin A1	+H	252.88	±	0.11	0.05
Enniatin A1	+K	258.50	±	0.16	0.06
Enniatin A1	+NH4	259.61	±	0.15	0.06
Enniatin B	+H	243.67	±	0.05	0.02
Enniatin B	+Na	248.96	±	0.15	0.06
Enniatin B	+NH4	250.47	±	0.19	0.08
Enniatin B1	+H	248.35	±	0.07	0.03
Enniatin B1	+Na	254.89	±	0.20	0.08
Enniatin B1	+NH4	256.08	±	0.31	0.12
Fumonisin B1	+H	258.61	±	0.11	0.04
Fumonisin B1	+Na	262.30	±	0.58	0.22
Fumonisin B1	-H	262.47	±	0.02	0.01
Fumonisin B2	+H	259.59	±	0.22	0.09
Fumonisin B2	+Na	261.82	±	0.17	0.06
Fumonisin B2	-H	261.73	±	0.08	0.03
Fumonisin B3	+H	257.37	±	0.40	0.16
Fumonisin B3	+Na	260.93	±	0.91	0.35
Fumonisin B3	-H	260.62	±	0.10	0.04
Fusarenon X	+H	173.63	±	0.26	0.15
Fusarenon X	+K	184.70	±	0.33	0.18
Fusarenon X	+Na	184.26	±	0.27	0.15
Fusarenon X	-H	176.15	±	0.52	0.30
Fusarenon X	-CH2O - H	158.30	±	0.08	0.05
Gliotoxin	+Na	168.64	±	0.11	0.07
HT-2 Toxin	+H	192.40	±	0.51	0.26
HT-2 Toxin	+K	208.23	±	0.15	0.07
HT-2 Toxin	+Na	205.59	±	0.17	0.08
HT-2 Toxin	+NH4	206.67	±	0.41	0.20
Hydrolized FB1	+H	205.19	±	0.99	0.48
Hydrolized FB1	+K	216.53	±	0.07	0.03
Hydrolized FB1	+Na	212.73	±	0.11	0.05
Hydrolized FB1	+CH3COO	221.34	±	0.17	0.08
Hydrolized FB2	+H	205.79	±	1.45	0.70
Hydrolized FB2	+K	215.57	±	0.40	0.19
Hydrolized FB2	+Na	212.00	±	0.38	0.18
Hydrolized FB2	+CH3COO	218.35	±	0.06	0.03
Hydrolized FB3	+H	205.29	±	0.23	0.11
Hydrolized FB3	+K	214.14	±	0.19	0.09
Hydrolized FB3	+Na	209.21	±	0.28	0.13
Hydrolized FB3	+CH3COO	219.00	±	0.27	0.12
Mycophenolic acid	+H	165.78	±	0.18	0.11
Mycophenolic acid	+Na	175.71	±	0.20	0.12

Mycophenolic acid	-H	176.13	±	0.12	0.07
Neosolaniol	+K	186.65	±	0.21	0.11
Neosolaniol	+Na	184.22	±	0.13	0.07
Nivalenol	+H	162.33	±	0.17	0.11
Nivalenol	+Na	174.29	±	0.05	0.03
Nivalenol	-H	165.65	±	0.17	0.10
Nivalenol	+CH3COO	178.26	±	0.09	0.05
Nivalenol	-CH2O - H	160.67	±	0.12	0.08
Ochratoxin A	+H	187.66	±	0.06	0.03
Ochratoxin A	+K	193.83	±	0.25	0.13
Ochratoxin A	+Na	196.73	±	0.09	0.05
Ochratoxin A	-H	192.99	±	0.28	0.15
Patulin	-H	125.44	±	0.14	0.11
Roquefortine C	+H	197.11	±	0.22	0.11
Roquefortine C	-H	198.10	±	0.04	0.02
Sterigmatocystin	+H	165.05	±	0.27	0.16
Sterigmatocystin	+Na	182.96	±	0.25	0.14
T-2 Glucoside alpha	+K	248.32	±	0.13	0.05
T-2 Glucoside alpha	+Na	244.71	±	0.30	0.12
T-2 Glucoside alpha	-H	248.34	±	0.12	0.05
T-2 Glucoside alpha	+CH3COO	253.52	±	0.11	0.04
T-2 Glucoside beta	+K	253.47	±	0.18	0.07
T-2 Glucoside beta	+NH4	253.83	±	0.14	0.05
T-2 Glucoside beta	+Na	251.74	±	0.69	0.28
T-2 Glucoside beta	-H	249.05	±	0.58	0.23
T-2 Glucoside beta	+CH3COO	263.91	±	0.13	0.05
T-2 Toxin	+H	201.63	±	0.42	0.21
T-2 Toxin	+K	214.53	±	0.04	0.02
T-2 Toxin	+Na	212.33	±	0.11	0.05
T-2 Toxin	+NH4	213.36	±	0.32	0.15
Tentoxin	+H	198.73	±	0.12	0.06
Tentoxin	+K	200.18	±	0.15	0.07
Tentoxin	+Na	198.00	±	0.13	0.07
Tentoxin	-H	198.45	±	0.12	0.06
Zearalenol-alpha	+H	173.28	±	0.02	0.01
Zearalenol-alpha	+Na	178.26	±	0.16	0.09
Zearalenol-alpha	-H	178.43	±	0.05	0.03
Zearalenol-beta	+H	173.16	±	0.09	0.05
Zearalenol-beta	+Na	179.59	±	0.19	0.11
Zearalenol-beta	-H	178.03	±	0.11	0.06
Zearalenol-beta	+CH3COO	192.23	±	0.39	0.20
Zearalenone	+H	172.50	±	0.10	0.06
Zearalenone	+Na	177.09	±	0.13	0.07
Zearalenone	-H	176.68	±	0.07	0.04
Zearalenone	+CH3COO	189.78	±	0.14	0.08
Zearalenone-14-glucoside	+H	218.89	±	1.48	0.68
Zearalenone-14-glucoside	+K	213.23	±	0.87	0.41
Zearalenone-14-glucoside	+Na	210.26	±	0.41	0.20
Zearalenone-14-glucoside	-Glc	177.00	±	0.08	0.05
Zearalenone-14-glucoside	-Glc	175.63	±	0.12	0.07
Zearalenone-16-glucoside	-H	219.30	±	0.11	0.05

Table S3. Composition of CCS calibration solution used for positive ion mode.

Compound	Positive ionization mode	
	m/z	CCS (Å^2)
Acetaminophen	152.0706	130.4
Reserpine fragment	195.0877	138.2
Sulfaguanidine	215.0597	146.8
Sulfadimethoxine	311.0809	168.4
Val-Tyr-Val	380.2180	191.7
Verapamil	455.2904	208.8
Terfenadine	472.3210	228.7
Polyalanine	516.2776	211.0
Leucine Enkephalin	556.2766	229.8
Polyalanine	587.3148	252.3
Reserpine	609.2807	252.3
Polyalanine	658.3519	243.0
Polyalanine	729.3890	256.0
Polyalanine	800.4261	271.0
Polyalanine	871.4632	282.0
Polyalanine	942.5003	294.0
Polyalanine	1013.5374	306.0
Polyalanine	1084.5746	321.5
Polyalanine	1155.6117	333.6
Ultramark 1621	1022.0034	263.1
Ultramark 1621	1121.9970	276.5
Ultramark 1621	1221.9843	291.2
Ultramark 1621	1321.9843	304.0
Ultramark 1621	1421.9779	316.7
Ultramark 1621	1521.9715	329.0
Ultramark 1621	1621.9651	340.1
Ultramark 1621	1721.9587	351.3
Ultramark 1621	1821.9523	362.1
Ultramark 1621	1921.9459	372.6

Table S4. Composition of CCS calibration solution used for negative ion mode

Compound	Negative ionization mode	
	m/z	CCS (Å^2)
Acetaminophen	150.0561	131.5
Theophylline	179.0575	132.4
Sulfaguanidine	213.0452	145.2
Sulfadimethoxine	309.2034	170.1
Val-Tyr-Val	378.2034	192.5
Leucine Enkephalin	554.2620	225.3
Perfluoroheptanoic acid-CO2	318.9766	130.1
Perfluorooctanoic acid-CO2	368.9766	137.2
Polyalanine	585.3002	227.7
Reserpine	607.2661	265.2
Polyalanine	656.3373	242.1
Polyalanine	727.3744	255.9
Polyalanine	798.4115	268.5
Polyalanine	869.4487	280.2
Polyalanine	940.4856	294.6
Polyalanine	1011.5288	308.8
Polyalanine	1082.5600	322.4
Ultramark 1621	1165.9880	275.8
Ultramark 1621	1265.9816	288.0
Ultramark 1621	1365.9752	299.7
Ultramark 1621	1465.9688	311.7
Ultramark 1621	1565.9624	323.7
Ultramark 1621	1665.9560	334.7
Ultramark 1621	1765.9496	346.2
Ultramark 1621	1865.9432	357.1
Ultramark 1621	1965.9369	367.2

Table S5. Theoretical CCS (Å²) for mycotoxins obtained using AllCCS and CCSbase prediction models.

Mycotoxin	Adduct	TWIMS CCS (Å²)	MetCCS_prediction	CCSbase_prediction
15-Acetyldeoxynivalenol	[M+Na]+	176.8	180.8	181.6
15-Acetyldeoxynivalenol	[M+H]+	169.5	177.3	176.7
15-Acetyldeoxynivalenol	[M-H]-	176.5	182.3	188.6
3-Acetyldeoxynivalenol	[M+Na]+	184.4	181.2	181.6
3-Acetyldeoxynivalenol	[M+NH4]+	179.9	180.5	185.6
3-Acetyldeoxynivalenol	[M+H]+	170.2	177.8	176.7
3-Acetyldeoxynivalenol	[M-H]-	180.0	182.4	188.6
3-Acetyldeoxynivalenol	[M+K]+	186.6	N.A.	178.2
Aflatoxin B1	[M+Na]+	178.8	174.3	168.6
Aflatoxin B1	[M+H]+	163.8	170.2	161.6
Aflatoxin B1	[M-H]-	171.9	175.5	170
Aflatoxin B1	[M+K]+	177.8	N.A.	177.2
Aflatoxin B2	[M+Na]+	180.2	174.6	168.6
Aflatoxin B2	[M-H]-	174.0	177.0	170.9
Aflatoxin B2	[M+H]+	165.3	170.5	162.5
Aflatoxin B2	[M+K]+	179.4	N.A.	177.2
Aflatoxin G1	[M+Na]+	181.7	177.2	176.4
Aflatoxin G1	[M-H]-	174.9	177.6	175.7
Aflatoxin G1	[M+H]+	165.8	173.1	167.1
Aflatoxin G1	[M+K]+	180.9	N.A.	180.3
Aflatoxin G2	[M+Na]+	183.3	177.4	176.3
Aflatoxin G2	[M-H]-	176.9	179.2	176.5
Aflatoxin G2	[M+H]+	168.1	173.4	167.9
Aflatoxin G2	[M+K]+	182.9	N.A.	180.2
Aflatoxin M1	[M+Na]+	181.2	177.2	172.5
Aflatoxin M1	[M-H]-	173.1	177.5	172.7
Aflatoxin M1	[M+H]+	166.7	173.3	166.5
Aflatoxin M1	[M+K]+	180.2	N.A.	178.4
Alternariol	[M+Na]+	173.1	160.6	167.2
Alternariol	[M+H]+	151.5	155.9	153.6
Alternariol	[M-H]-	151.6	157.7	155.1
Alternariol-methylether	[M+Na]+	176.5	164.6	171.4
Alternariol-methylether	[M+H]+	154.3	160.0	157.4
Alternariol-methylether	[M-H]-	156.8	163.6	160
Beauvericin	[M+H]+	282.4	291.2	279.7
Beauvericin	[M+Na]+	288.8	291.8	287.3
Beauvericin	[M+NH4]+	290.2	291.7	291.9
Beauvericin	[M-H]-	277.5	261.1	288.2
Beauvericin	[M+K]+	289.8	N.A.	290.4
Citrinin	[M-H]-	158.0	158.7	153.5

Citrinin	[M+Na]+	167.0	160.4	164
Citrinin	[M+H]+	150.3	155.9	152.8
Citrinin	[M+K]+	164.4	N.A.	163.1
Cyclopiazonic acid	[M+Na]+	187.0	183.7	183.2
Cyclopiazonic acid	[M+H]+	173.7	180.0	178.4
Deoxynivalenol	[M+Na]+	171.5	172.1	171.3
Deoxynivalenol	[M+H]+	160.8	168.2	165.8
Deoxynivalenol	[M-H]-	170.3	172.4	177.6
Deoxynivalenol	[M+K]+	173.3	N.A.	166.9
Diacetoxyscirpenol	[M+H]+	179.2	185.4	185.4
Diacetoxyscirpenol	[M+Na]+	180.9	188.5	189.7
Diacetoxyscirpenol	[M+NH4]+	183.0	187.8	194.1
Diacetoxyscirpenol	[M+K]+	183.3	N.A.	187.4
DON-3-glucoside	[M+H]+	209.7	205.4	197.7
DON-3-glucoside	[M+Na]+	205.6	207.7	199.3
DON-3-glucoside	[M+NH4]+	210.6	207.2	205.3
DON-3-glucoside	[M-H]-	208.4	204.6	209.7
DON-3-glucoside	[M+K]+	209.4	N.A.	199.8
Enniatin A	[M+NH4]+	262.6	260.6	259.6
Enniatin A	[M+Na]+	261.2	260.7	261.1
Enniatin A	[M+H]+	255.5	260.3	256.3
Enniatin A	[M+K]+	261.6	N.A.	261.4
Enniatin A1	[M+NH4]+	258.0	256.8	256.3
Enniatin A1	[M+H]+	251.5	256.5	252.3
Enniatin A1	[M+Na]+	256.6	256.9	257.9
Enniatin B	[M-H]-	249.8	237.0	248.4
Enniatin B	[M+NH4]+	248.9	247.7	249.5
Enniatin B	[M+H]+	242.0	247.3	244.3
Enniatin B	[M+Na]+	247.0	247.7	249.7
Enniatin B1	[M+NH4]+	254.7	252.5	252.9
Enniatin B1	[M+Na]+	253.3	252.6	253.3
Enniatin B1	[M+H]+	248.1	252.2	248.3
Fumonisin B1	[M+Na]+	261.2	268.6	263.7
Fumonisin B1	[M+H]+	256.2	268.0	260.4
Fumonisin B1	[M-H]-	262.5	268.9	269.8
Fumonisin B1	[M+K]+	261.3	N.A.	267
Fumonisin B2	[M+H]+	258.4	265.3	257.9
Fumonisin B2	[M+Na]+	262.1	265.9	261.7
Fumonisin B2	[M-H]-	261.5	264.2	261.7
Fumonisin B2	[M+K]+	259.8	N.A.	264.4
Fumonisin B3	[M+Na]+	260.7	266.0	261.7
Fumonisin B3	[M+H]+	256.7	265.4	257.9
Fumonisin B3	[M-H]-	261.7	264.2	265.7
Fumonisin B3	[M+K]+	260.3	N.A.	264.4
Fusarenon X	[M+Na]+	185.6	183.8	183.1
Fusarenon X	[M+NH4]+	183.5	183.0	187.4
Fusarenon X	[M+H]+	174.6	180.5	179
Fusarenon X	[M-H]-	181.9	184.8	189.9
Fusarenon X	[M+K]+	186.1	N.A.	180.4
Gliotoxin	[M-H]-	164.7	171.1	172.3
Gliotoxin	[M+Na]+	168.2	175.2	179.8

Gliotoxin	[M+H]+	161.1	171.6	174.5
HT-2 Toxin	[M+Na]+	206.9	201.5	200.7
HT-2 Toxin	[M+NH4]+	209.3	200.9	205.8
HT-2 Toxin	[M+H]+	196.4	199.0	198.3
HT-2 Toxin	[M+K]+	209.8	N.A.	200.3
Hydrolized FB1	[M-H]-	211.8	205.4	202.7
Hydrolized FB1	[M+Na]+	213.2	218.1	206.7
Hydrolized FB1	[M+H]+	206.6	215.6	208.8
Hydrolized FB1	[M+K]+	216.7	N.A.	214.1
Hydrolized FB2	[M-H]-	209.7	202.7	201.8
Hydrolized FB2	[M+Na]+	212.3	215.4	205.7
Hydrolized FB2	[M+H]+	205.7	212.8	206.5
Hydrolized FB2	[M+K]+	215.8	N.A.	209.2
Hydrolized FB3	[M-H]-	208.1	202.7	201.8
Hydrolized FB3	[M+Na]+	209.3	215.3	205.7
Hydrolized FB3	[M+H]+	207.0	212.7	206.5
Hydrolized FB3	[M+K]+	214.3	N.A.	209.2
Meleagrin	[M+Na]+	209.5	204.7	206.9
Meleagrin	[M+H]+	201.7	201.8	200.9
Meleagrin	[M-H]-	199.5	204.3	200.4
Mycophenolic acid	[M-H]-	176.3	178.9	172.9
Mycophenolic acid	[M+Na]+	176.1	179.0	179.5
Mycophenolic acid	[M+H]+	167.0	175.2	173
Mycophenolic acid	[M+K]+	178.6	N.A.	182.9
Neosolaniol	[M+H]+	184.2	188.0	187.6
Neosolaniol	[M+Na]+	184.2	191.0	191.2
Neosolaniol	[M+NH4]+	186.4	190.3	195.8
Neosolaniol	[M+K]+	186.4	N.A.	189.4
Nivalenol	[M+Na]+	175.7	175.0	172.9
Nivalenol	[M-H]-	175.3	174.7	178.8
Nivalenol	[M+NH4]+	173.5	174.2	177.1
Nivalenol	[M+H]+	163.9	171.2	168.1
Nivalenol	[M+K]+	177.1	N.A.	169.1
Ochratoxin A	[M-H]-	193.7	190.7	193.5
Ochratoxin A	[M+Na]+	198.2	195.1	198.7
Ochratoxin A	[M+H]+	188.1	192.1	189.5
Ochratoxin A	[M+K]+	201.4	N.A.	204.6
Patulin	[M+H]+	128.2	131.3	126.4
Patulin	[M-H]-	123.6	127.1	128.5
Paxilline	[M+H]+	225.1	207.2	206.8
Paxilline	[M+Na]+	227.0	209.9	210.2
Paxilline	[M-H]-	214.2	213.4	210.5
Paxilline	[M+K]+	225.1	N.A.	214.9
Penicillic acid	[M+Na]+	144.6	143.2	143.6
Penicillic acid	[M+H]+	132.4	138.5	135.3
Penitrem A	[M+Na]+	263.3	241.5	251.5
Phomopsin A	[M+Na]+	268.9	268.9	241.1
Phomopsin A	[M-H]-	262.5	257.2	235.7
Phomopsin A	[M+H]+	263.4	268.8	239.9
Roquefortine C	[M+H]+	198.9	194.7	193
Roquefortine C	[M-H]-	199.1	195.2	193.3

Stachybotrylactam	[M+Na]+	219.4	196.2	198.4
Stachybotrylactam	[M-H]-	200.3	199.7	197.3
Stachybotrylactam	[M+H]+	197.3	193.2	194.7
Sterigmatocystin	[M+Na]+	184.7	178.2	178.7
Sterigmatocystin	[M+H]+	165.3	174.0	168.4
Sterigmatocystin	[M+K]+	184.4	N.A.	185.2
T-2 Glucoside alpha	[M-H]-	246.1	230.6	232.8
T-2 Glucoside alpha	[M+NH4]+	246.0	237.7	239.2
T-2 Glucoside alpha	[M+Na]+	242.1	237.9	235.9
T-2 Glucoside alpha	[M+H]+	244.6	237.0	240.8
T-2 Glucoside alpha	[M+K]+	245.2	N.A.	243.8
T-2 Glucoside beta	[M-H]-	249.8	230.6	232.8
T-2 Glucoside beta	[M+NH4]+	251.9	237.7	239.2
T-2 Glucoside beta	[M+Na]+	247.7	237.9	235.9
T-2 Glucoside beta	[M+H]+	242.2	237.0	240.8
T-2 Glucoside beta	[M+K]+	249.6	N.A.	243.8
T-2 Toxin	[M+Na]+	212.9	209.4	208.7
T-2 Toxin	[M+NH4]+	214.4	209.0	213.9
T-2 Toxin	[M+H]+	202.4	207.4	207
T-2 Toxin	[M+K]+	215.7	N.A.	209.5
Tentoxin	[M-H]-	199.5	199.1	195.3
Tentoxin	[M+H]+	198.3	201.5	195.2
Tentoxin	[M+Na]+	199.0	204.4	205.1
Tentoxin	[M+K]+	200.3	N.A.	206.9
Tenuazonic acid	[M-H]-	144.1	145.7	142
Tenuazonic acid	[M+Na]+	151.8	148.7	152.4
Tenuazonic acid	[M+H]+	141.6	143.9	144.4
Verrucarol	[M+Na]+	171.7	167.5	167.7
Verrucarol	[M+H]+	157.2	163.3	161.4
Verrucarol	[M+NH4]+	165.4	166.6	171.9
Verrucarol	[M+K]+	170.6	N.A.	162.7
Verruculogen	[M+Na]+	235.4	219.7	231.3
Verruculogen	[M-H]-	230.2	220.5	230.8
Zearalenol-alpha	[M-H]-	179.3	183.5	172.9
Zearalenol-alpha	[M+H]+	174.0	179.9	168.7
Zearalenol-alpha	[M+Na]+	179.7	184.1	175.9
Zearalenol-alpha	[M+K]+	182.2	N.A.	181
Zearalenol-beta	[M-H]-	178.7	183.5	172.9
Zearalenol-beta	[M+H]+	174.0	179.9	168.7
Zearalenol-beta	[M+Na]+	181.0	184.1	175.9
Zearalenol-beta	[M+K]+	182.1	N.A.	181
Zearalenone	[M+H]+	172.5	178.5	166.7
Zearalenone	[M-H]-	177.2	182.6	171.3
Zearalenone	[M+Na]+	178.3	182.7	174.7
Zearalenone	[M+K]+	178.8	N.A.	179.2
Zearalenone-14-glucoside	[M-H]-	222.1	208.3	211
Zearalenone-14-glucoside	[M+Na]+	215.8	216.7	210
